# Analysis of Sociodemographic and Psychological Variables Involved in Sleep Quality in Nurses

**DOI:** 10.3390/ijerph16203846

**Published:** 2019-10-11

**Authors:** María del Carmen Pérez-Fuentes, María del Mar Molero Jurado, María del Mar Simón Márquez, José Jesús Gázquez Linares

**Affiliations:** 1 University of Almería, 04120 Almería, Spain; mpf421@ual.es (M.d.C.P.-F.); msm112@ual.es (M.d.M.S.M.); 2 Universidad Politécnica y Artística del Paraguay, 1628 Asunción, Paraguay; 3 Universidad Autónoma de Chile, 4780000 Santiago, Chile; jlinares@ual.es

**Keywords:** sleep quality, nurses, related factors, mediation

## Abstract

Background: Sleep quality is related to health and quality of life and can lead to the development of related disorders. This study analyzed the sociodemographic and psychological factors related to sleep quality in nurses. Methods: The sample comprised 1094 nurses who were assessed according to the Pittsburgh Sleep Quality Index, the Rosenberg Self-esteem Questionnaire, the Goal Content for Exercise Questionnaire, the Brief Emotional Intelligence Inventory, and the Three-Factor Eating Questionnaire-R18. Results: The results confirm the impacts of diet, motivation for physical exercise, emotional intelligence, and overall self-esteem on sleep quality in nurses. Conclusions: Sleep quality in healthcare professionals is vitally important for performance at work; therefore, appropriate strategies should be applied to improve it.

## 1. Introduction

Sleep quality is related to health and quality of life. Negative factors involved in poor sleep quality are directly associated with worse health, increased risk of mortality, bodily changes that require healthcare, as well as an increase in the proportion of people suffering psychological disorders such as depression [1]. There are various factors involved in sleep quality, including age, physical condition, self-esteem, type of work, and poor diet, among others [2].

In the nursing profession, sleep quality is essential for giving proper care to patients. Nursing also requires high levels of concentration, the carrying out of complex tasks, and huge responsibilities. Factors such as the pace of work, overtime, and shift work may produce stress, which may increase the probability of suffering from sleep disorders [3,4,5].

Various studies have shown how shift work can negatively impact nurses’ sleep quality. A high proportion of nurses report having sleep disorders, stress, and physical and mental exhaustion due to the shift patterns and rotations they work [6,7,8,9,10]. Lack of sleep can have an impact on healthcare practice, increasing the likelihood of errors. Nurses’ health and the quality of the care they give patients is influenced by poor quality sleep, often linked to working shifts in hospitals [11].

### 1.1. Related Factors Influencing Sleep Quality

Nurses’ sleep quality may be related to various factors. Shift work has been linked to a series of negative consequences, such as the alteration of the circadian rhythm, the decrease in sleep hours, as well as the increase in fatigue [12] All of this can increase the risk of mistakes and accidents that may affect patient safety [13]. On the other hand, sleep disturbance associated with shift work has an impact on the health of professionals, so various studies relate working in shifts to serious health disorders, such as metabolic disorders, cardiovascular diseases, and psychological disorders, decreasing the quality of life of workers [14,15,16].

The probability of suffering from a sleep disorder increases with age by 20%–30% [2,17], which may mean a reduction in sleep quantity and quality in over 50-year-olds, who may have difficulties falling asleep or staying asleep during the night. Other age-related factors may include the homeostatic and circadian rhythms, reduced efficacy of sleep, and the length of daytime naps [18,19]. All of this seems to show how sleep interruption and not sleeping have adverse consequences on health, accelerating premature ageing [20,21,22], which can influence cognitive impairment, as the tasks that assess working memory, attention and executive function, among others are affected [23].

Generally, women encounter greater obstacles to falling asleep [24]. They have a higher risk of suffering from insomnia, whereas men tend to sleep better, with fewer difficulties when it comes to falling asleep [25].

Another factor to consider in terms of sleep quality is marital status. On various occasions, it has been shown that people who are divorced or married have poorer quality sleep than those who are single. To a large extent, this may be due to the stress of reconciling work and home life [26]. Thus, difficulty sleeping can be related to an increased risk of having a low marital quality, since people who suffer from sleep disorders experience great irritability and low tolerance, which usually leads to conflicts in the couple, and in a negative and volatile mood in the couple [27].

According to recent research, sleep disturbance is related to emotional intake. Emotional intake responds to a set of negative emotions, such as anxiety, depression, anger, among others, due to low self-esteem and other stressors [9]. On several occasions, emotional intake has been associated with binge eating, that is, people eat based on their negative emotional state [28]. Sleep modulates emotions and affective neuronal systems, which is why lack of sleep leads to uncontrolled food intake often caused by chronic emotional stress resulting from lack of sleep [29].

Physical inactivity may bring with it serious health problems such as diabetes, hypertension, obesity, heart disease, and sleep disorders. This makes physical exercise one of the factors related to sleep quality [30].

Generally, doing frequent physical exercise is beneficial for improving sleep, both in terms of sleep latency and slow-wave sleep. These types of benefits are usually seen in adults who are middle-aged and older who have difficulties falling asleep [31].

On various occasions, nurses face high-stress situations and situations of emotional and physical exhaustion, which are linked to a deterioration in health [32,33], and which can contribute to low levels of emotional intelligence and may cause sleep disorders as well as, and as a consequence of, poor sleep quality. Emotional intelligence is essential for understanding people’s psychosocial adjustment [34,35,36] and for facing life’s continual challenges, in the workplace and in one’s health [37,38,39]. People with high levels of emotional intelligence and appropriate control of their emotions exhibit less exhaustion and depersonalization, as well as better sleep quality [40,41].

Similarly, low levels of emotional intelligence may result in various emotional disorders, mood disorders, and stress and anxiety, producing sleep disturbances or insomnia [42,43]. Regarding the association between stress and sleep disturbances, several studies show how stress management is related to sleep disorders and its quality, which is why proper stress management increases sleep quality due to a decrease in anxiety and work distress, as well as a reduction in negative health consequences [44,45]. Poor stress management is associated with physical and mental health risk factors related to anxiety, depression, and sleep disorders [44]. A decrease in the hours of sleep, as well as the interruption of the circadian rhythm, increase stress and cause the hypothalamus–pituitary–adrenal system to be activated constantly, thus annulling the control of the circadian rhythm and interrupting it [46].

Night-time sleep is vital for physical and mental wellbeing. Sleeping less than seven or eight hours a day is associated with negative health consequences and psychological problems, such as low self-esteem [47,48]. An increase in sleep duration entails better self-esteem and higher optimism, which translates to being determinants of good health [49]. In addition, nurses exhibiting high self-esteem and better patient care are less likely to suffer from exhaustion during their working day [50].

On similar lines, poor quality and small amounts of sleep reduce an individual’s optimism as well as their self-esteem. Low levels of self-esteem indicate high levels of vulnerability [51,52] caused by poor management of the stress generated by shift work with inflexible rotations, work overload, increased health demand, workplace conflicts, and the emotional burden that may lead to depression [53,54,55]. Depression can begin during nursing studies, due to the stress and anxiety that labor incorporation can generate after the end of their studies. As such, being in a profession with a great emotional load can generate stress and exhaustion that can be externalized before joining the world of work [56], which may lead to poor sleep quality in sufferers [53].

### 1.2. Current View of Work

A review of the literature on this subject highlighted the need to analyze the factors related to sleep quality in healthcare workers in relation to factors such as age, sex, diet, physical exercise, emotional intelligence, and self-esteem. This current research aims to present new analytical data on the influence of self-esteem, emotional eating, and age on sleep quality in nurses and the implications of that on professional practice.

Following on from the above theoretical evidence, we consider the following hypotheses: 

H1: Sleep quality is related to age, so that the older one is, the worse the quality of sleep is.

H2: The quality of sleep does not differ significantly between men and women.

H3: The quality of sleep has different values according to the marital status group.

H4: Self-esteem, emotional eating, and emotional intelligence components are variables related to sleep quality in nurses.

H5: There are differences in terms of the variables associated with the quality of sleep in nurses, according to marital status.

H6: Low self-esteem and an emotional eating style (mediating function) is related to poor sleep quality in nurses.

H7: Stress management (IE) is a mediator in the relationship between low self-esteem and poor sleep quality in nurses.

H8: Age is a moderator of the indirect effects of self-esteem, emotional eating, and stress management on sleep quality in nurses.

## 2. Materials and Methods 

### 2.1. Participants

The sample was selected by random sampling in different health institutions. Voluntary collaboration was requested by email. The questionnaires were completed through a web platform in which the participants did not reveal their identification data. The sample comprised a total of 1094 nurses from the autonomous community of Andalucia (Spain), aged between 22 and 57 years old, mean age 32.40 (*SD* = 7.27). A minority (14.9%; *n* = 163) were men and 85.1% (*n* = 931) were women. Half (50.5%; *n* = 553) were single, while 41.7% (*n* = 515) were married or in a stable partnership, and 2.2% (*n* = 24) were divorced or separated, with 0.2% (*n* = 2) being widowed. Regarding the labor situation of the participants, at the time of data collection, 32.1% (*n* = 351) had a stable employment contract, 59.3% (*n* = 649) in a discontinuous employment situation, and the remaining 8.6% (*n* = 94) were unemployed.

### 2.2. Instruments

We created a questionnaire ad hoc to collect sociodemographic data. It was made up of 4 items with different questions to gather detailed data on the different variables. The variables considered for this study were: age, sex, and marital status.

The *Pittsburgh Sleep Quality Index* (PSQI) [57]. This consists of a total of 24 items for measuring subjects’ sleep quality. There are 19 self-applied questions and 5 questions to be evaluated by the patients’ partner or roommate. Only the self-applied questions were used in the score in this study. The 19 items combine together to form seven scoring components, each of which has a range of 0 to 3. A score of 0 indicates low difficulty and a score of 3 indicates severe difficulty. In this questionnaire, a total score of 5 or more indicates poor sleep quality [58]. The seven component scores are added together to give an overall PSQI score, which can range from 0 to 21 with higher scores indicating poor sleep quality. In the current study the internal consistency was calculated as α = 0.77.

The *Rosenberg Self-esteem Questionnaire* is made up of 10 items [59,60]. It is based on a 4-point Likert-type scale where 1 = completely agree, 2 = agree, 3 = disagree, and 4 = totally disagree. The internal consistency was α = 0.82.

The *Goal Content for Exercise Questionnaire (GCEQ)* from [61] is composed of 20 items in total, grouped into five factors with four items each: social affiliation, image, health, social recognition, and development of skills. It uses a Likert scale from 1 (not important at all) to 7 (extremely important). In this study the internal consistency was α = 0.92.

The *Brief Emotional Intelligence Inventory (EQ-I-M20)* [62]. This is composed of 20 items using a 4-point Likert-type scale with 5 subscales: intrapersonal, interpersonal, stress management, Adaptability, and Mood. The instrument determines the stable feelings of personal competence to effectively face a wide variety of stressful situations. In our study the internal consistency was α = 0.87.

The *Three-Factor Eating Questionnaire (R18)* [63]. This questionnaire is made up of 18 items. It measures three aspects of eating behavior. It uses a 4-point response scale (absolutely true: 1, mostly true: 2, mostly false: 3, and absolutely false: 4) and the elements are totaled for scores in subscales: restricted eating, uncontrolled eating, and emotional eating. The coefficient for internal consistency was α = 0.87.

### 2.3. Procedure

Data was collected by carrying out a series of tests with nurses who voluntarily cooperated for this study. They were given the relevant information about this research and the participants were asked to answer the questions sincerely and were assured of absolute confidentiality. The questionnaire was administered individually online and included control questions to detect random responses. We have protected the participants anonymity in this study, thus complying with research ethics. This study was approved by the Bioethics Committee of the University of Almería.

### 2.4. Data Analysis

Firstly, we performed a descriptive and comparative analysis of means, taking the overall score of the sleep quality index as the dependent variable and looking at the sample sociodemographic variables (sex and marital status) using the Student t test and analysis of variance, respectively. We examined the relationship between sleep quality and other sociodemographic variables, such as age, by applying a bivariate correlation using the Pearson coefficient. Confidence intervals were estimated at 95%.

In order to identify the factors related to sleep quality, we calculated the Pearson correlation coefficient as well as the corresponding descriptive statistics. Finally, in order to understand how the implicated variables (eating, motivation to do physical exercise, emotional intelligence, and self-esteem) were related to the criterion variable, we carried out a stepwise multiple linear regression analysis. Subsequently, based on the results obtained, simple mediation analysis for the sleep quality index was computed, taking as the independent variable the one that had the greatest explanatory weight in the model, and including as potential mediators the psychological variables that were also involved in the regression equation and which obtained a significant weight. In addition, in order to verify the existence of possible moderation effects, moderate mediation analysis was conducted. To do this, PROCESS was used, a macro for SPSS developed by researchers in [64]. We used 5000 bootstrap samples to generate 95% bias-corrected confidence intervals (CI) for both direct and indirect effects.

## 3. Results

### 3.1. Sleep Quality in Nurses and Sociodemographic Variables

The sample as a whole scored a mean of 6.44 (standard deviation (*SD*) = 2.90) in the overall sleep quality index. Looking at the sociodemographic variables, there was a positive correlation between sleep quality and age (*r* = 0.13, *p* < 0.001, 95% CI (.071, 0.188)). No statistically significant differences (*t* = −0.13; *p* = 0.89) were found between men (*M* = 6.42; *SD* = 2.80) and women (*M* = 6.45; *SD* = 2.92).

We did see significant differences in sleep quality in terms of marital status (*F* = 5.92; *p* < 0.01). *Post hoc* testing indicated that single nurses (*M* = 6.21; *SD* = 2.84) exhibited better quality sleep compared to divorced or separated nurses (*M* = 8.13; *SD* = 3.43). The married or with a stable partner obtained a mean of 6.64 (*SD* = 2.89), although they did not present statistically significant differences when compared with the rest of the marital status groups.

### 3.2. Factors Associated with Sleep Quality in Nurses

The correlation coefficients indicated positive correlations between sleep quality and some of the dimensions associated with eating, motivation to do physical exercise, emotional intelligence, and overall self-esteem.

Table 1 shows positive correlations between the score for sleep quality and scores in uncontrolled eating (*r* = 0.17; *p* < 0.001) and emotional eating (*r* = 0.19; *p* < 0.001). In other words, nurses demonstrated worse sleep (higher scores in the overall PSQI index) as they tended to habitually engage in either of these two types of eating behaviors.

In motivation to do physical exercise, it was Image that maintained a positive correlation with sleep quality (*r* = 0.06; *p* < 0.05). The other factors (social affiliation, managing health, social recognition, and development of skills) did not correlate with sleep quality.

With respect to the components of emotional intelligence, as shown in Table 1, the sleep quality index was negatively correlated with stress management (*r* = −0.14; *p* < 0.001), adaptability (*r* = −0.06; *p* < 0.05), and mood (*r* = −0.20; *p* < 0.001).

Finally, the correlation coefficient for overall self-esteem and the sleep quality index (*r* = −0.23; *p* < 0.001) shows that high scores in self-esteem are related with lower scores in the overall PSQI index, or better quality of sleep.

### 3.3. Related Variables to Sleep Quality in Nurses

According to the data shown in Table 2, the regression analysis produced four models, with model 4 having the best explanatory capacity, with 9.6% (*R*^2^ = 0.096) of the variance explained by the factors included in the model, the value of the effective size being *f*^2^ = 0.11. In order to confirm the model’s validity, we analyzed the independence of the residuals. The Durbin–Watson *D* statistic was *D* = 1.91, which confirmed the absence of positive and negative autocorrelation.

Table 2 shows that the value of *t* was associated with a probability of error of less than 0.05 in all of the variables included in the model. Furthermore, the standardized coefficients showed that the variables exhibiting greatest explanatory weight were self-esteem, emotional eating, and age.

Finally, the high values of tolerance indicators and low values of variance inflation factor (VIF) indicated the absence of collinearity between the variables in the model.

### 3.4. Related Variables to Sleep Quality in Nurses, According to Marital Status

Once significant differences in sleep quality were identified between the groups based on marital status, multiple linear regression models were applied for each of the categories (Table 3).

Regression analysis was carried out for the category “marital status = divorced or separated” with three models, the third of which explained 64.1% of the variance (*R*^2^ = 0.641). The size of the effective value was *f*^2^ = 1.78. The absence of positive and negative autocorrelation was confirmed by the Durbin–Watson *D* statistic (*D* = 2.34). The value of *t* was associated with a probability of error of less than 0.05 and the standardized coefficients showed that the variable with greatest explanatory weight was emotional eating. The values of tolerance indicators and VIF close to 1 indicated the absence of collinearity.

For the category “marital status = single”, the regression analysis produced two models. The second model explained 7% of the variance (*R*^2^ = 0.070), with collinearity absent (*D* = 1.98). The size of the effective value was *f*^2^ = 0.07. The value of *t* was associated with a probability of error of less than 0.05 in both variables in the model (overall self-esteem and age), with self-esteem demonstrating more explanatory weight. The values of tolerance indicators and VIF close to 1 indicated the absence of collinearity.

Finally, for the category “marital status = married or in stable relationship”, Table 3 shows that the regression analysis resulted in three models. The third model demonstrated the most explanatory capacity, with 10.7% (*R*^2^ = 0.107) of the variance explained by the included factors, with collinearity absent (*D* = 1.88), and the value for the effective size *f*^2^ = 0.11. In this case, the value of *t* was associated with a probability of error of less than 0.05 in two of the variables included in the model (mood and uncontrolled eating), but not in stress management (*p* = 0.05). The standardized coefficients show mood to be the variable with greatest explanatory weight in the model. The values of tolerance indicators and VIF indicate the absence of collinearity.

### 3.5. Effects of Mediation and Moderate Medication on the Relationship between Self-Esteem and Sleep Quality

We start from the results obtained in the previous regression analyzes, where self-esteem is presented as the variable with the greatest explanatory weight in the equation. That is why it is considered as an independent variable in the mediation models that follow. As a dependent variable, in both cases, the quality of sleep (remember, after the interpretation of results, that a higher score in this index implies a worse quality of sleep).

**Figure 1:**
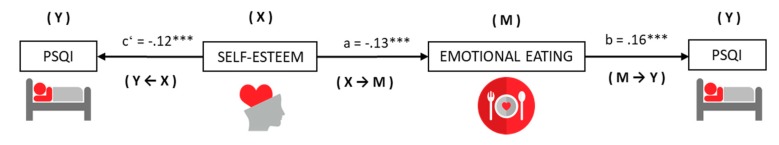
Mediation model of emotional intake in the relationship between self-esteem and sleep quality.

In the first model, the emotional eating variable is proposed as a mediator. As can be seen in Figure 1, of the simple linear regression analysis between the independent variable (X) and the mediating variable (M), self-esteem is presented as a significant variable within the model (B = −0.13, p < 0.001). On the other hand, Emotional intake (M) and self-esteem (X) show significant direct effects on sleep quality (Y): Emotional intake (B = 0.16, p < 0.001) and self-esteem (B = −0.12, p < 0.001). The total effect of the model is B = −0.14, p < 0.001. On the other hand, the analysis of indirect effects, based on the bootstraping procedure was significant for the indirect effect of self-esteem through emotional intake (B = −0.022, SE = 0.005, 95% CI (−0.034, −0.012)).

**Figure 2:**
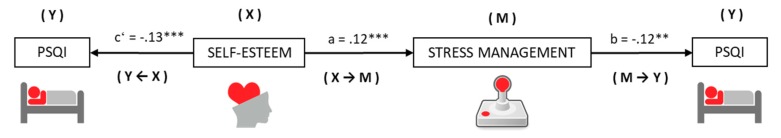
Mediation model of stress management in the relationship between self-esteem and sleep quality.

Figure 2 shows the results of the simple mediation analysis, including stress management as a mediating variable in the equation. Significant effects of self-esteem on stress management are observed (B = 0.12, p < 0.001), and also on the PSQI index (B = −0.12, p < 0.001). The model has a direct effect of Self-Esteem on the sleep quality index of B = −0.13, p < 0.001. Finally, with the analysis of the indirect effects (X → M → Y), through the bootstrapping technique, significant values are obtained (B = −0.015, SE = 0.005, 95% CI (−0.027, −0.006)).

Finally, taking into account that age was another of the variables that became part of the regression equation (Table 3), moderate mediation analyzes were carried out in order to verify the effects of age as a moderator (W) in each of the proposed mediation models (Figure 1 and Figure 2). For this, the age variable (<30 years and ≥30 years) was dichotomized.

First, in the mediation model of emotional intake in the relationship between self-esteem and sleep quality, age as a moderating variable has no statistically significant effect ((B = 1.03, SE = 1.107, 95% CI (−1.141, 3.204)), also the interaction term X * W ((B = −0.02, SE = 0.033, 95% CI (−0.092, 0.039)). Therefore, it can be found that the indirect effect is not moderated by age, with a Moderate Mediation Index of 0.35, SE = 0.005, 95% CI (−0.017, 0.006).

On the other hand, for the stress management mediation model in the relationship between self-esteem and sleep quality, age would not have a direct effect on the mediator ((B = 0.52, SE = 0.984, 95% CI (−1.404, 2.460)), in the same way as the interaction X * W ((B = −0.005, SE = 0.030, 95% CI (−0.064, 0.053)). Thus, with a moderate mediation index of 0.007, SE = 0.004, 95% CI (−0.007, 0.009), an association between the indirect effect and age as a moderating variable is not estimated.

## 4. Discussion

Following the analysis of the results, we identified a relationship between sleep quality and factors such as age, marital status, eating behavior, physical exercise, emotional intelligence, and self-esteem. In a similar way to the sample in the study by [17], it was found that as nurses get older, they sleep for less time and the quality of sleep deteriorates. In addition, poor sleep quality was associated with impaired cognitive function related to verbal memory, executive function, psychomotor speed, and attention [23].

In relation to the first hypothesis, a positive relationship between age and quality of sleep was shown: as age increases there is a greater difficulty in falling asleep or maintaining it throughout the night, consequently deteriorating the effectiveness of sleep [2,17,18,19].

Regarding the sex variable, no significant differences were found between both sexes, that is, the quality of sleep in men and women is similar. However, the studies of [24,25] indicate that it is women who claim to have a worse quality of sleep.

The data in this study demonstrate a direct relationship between sleep quality and marital status in all groups, confirming the findings from [26] that nurses who are married or divorced with children have worse quality sleep, owing to the need to balance work and family, which increases stress. On the other hand, it was observed that the married nurses show an explanatory capacity with respect to the state of mind compared to divorced or single nurses. This is because the people who suffer from sleep disturbances or insomnia are more susceptible to becoming irritated, which can generate conflicts in the relationship and a negative mood and alertness in the couple [27].

Similarly, eating behavior is linked to poor sleep quality in nurses, specifically, uncontrolled eating and emotional eating. These results are in line with previous research showing that uncontrolled eating can have direct negative effects on sleep quality generated by a series of negative emotions such as anxiety, depression, and anger [9,29].

Physical exercise is associated with sleep quality, especially in relation to the Image factor. However, the study by [30] found high levels in terms of social recognition and social support, in contrast to our findings.

We saw a negative relationship between sleep quality and the components of emotional intelligence. Conversely, the study by [9] and [43] noted that night shifts led to emotional disorders, emotional vulnerability, and stress in nurses and presented those as possible determinants of poor sleep quality.

In research by [49], high self-esteem, together with a good level of optimism, was a determinant of health. High scores in self-esteem are related to good sleep quality and beneficial for nursing performance [50].

Mediation models on the involvement of psychological variables in the relationship between self-esteem and sleep quality confirmed our hypothesis. First, low self-esteem is associated with the emotional intake derived from the anxiety and stress to which they are subjected [9,29], leading to an uncontrolled intake of junk food that consequently causes sleep disturbances [28]. On the other hand, low self-esteem is related to poor sleep quality, increasing their vulnerability, affecting their work performance, and reducing the quality of patient care [29].

Self-esteem was shown to be an essential factor to cope with the stress that nurses are subjected to, which is why low self-esteem can lead to poor stress management in which professionals do not feel qualified to face the challenges they face in their work environment, such as dealing with patients [44,52,54]. Thus, lack of sleep linked to low self-esteem is related to a deterioration of both physical and mental health [44].

Finally, age was shown to be a moderator in the mediation model of emotional intake in the relationship between self-esteem and sleep quality, as well as stress management, does not have a significant effect on the variables. The main health risks of nurses are associated with the work they do; most nurses suffer from sleep disorders arising from stress, fatigue, shift work, and the reconciliation of work with family and social life. Thus, nursing work can have consequences on the health and quality of life of nurses, increasing the risk of obesity, circulatory disorders, as well as increasing the likelihood of accidents in the workplace, derived on several occasions by low self-esteem and poor stress management. However, age is not decisive in these variables [16].

Some limitations of this study should be considered. Firstly, the overwhelming representation of women in the sample means that any generalization of the results must consider the sample characteristics, where there is a higher proportion of women than men in nursing. Secondly, the study focused on one of the branches of healthcare sciences, which could lead to bias if the results were generalized to other professions. In this study it is impossible to talk about a causal relationship, as it is a descriptive and cross-sectional study, but descriptive statistics can become, when we analyze the subject, more important than an experimental study. Besides, the strength of the relationships on the variables was moderated and the real interpretive value of the correlations must be considered. In the case of age, there is a direct effect, however, many other factors probably also contribute, given the generally small correlation, these should be studied in future work.

On the other hand, this study did not investigate the relationship that the use of internet and smart phones can have on sleep quality. Mobile phones are now part of people’s daily lives, which is why excessive use and addiction to mobile phones is a problem with respect to physical and psychological health, among which are sleep disruption and bad sleep quality [65].

The results of this study suggest future lines of research, increasing the sample size, and broadening the professions to other fields of healthcare. It would also be interesting to include other variables in the analysis, such as shift working, and self-efficacy, in relation to sleep disorders.

Intervention strategies may be developed from the results of this study, with the aim of improving sleep quality and quality of life for nurses and, in turn, improving patient care.

## 5. Conclusions

In general, it was observed that emotional intake and stress management are moderating variables of self-esteem and sleep quality; therefore, it is essential to propose intervention programs for stress management and emotional intake to achieve high self-esteem and consequently improve the quality of sleep in nursing professionals. Good quality sleep is essential for nurses to be able to do their jobs well and efficiently, as well as to improve their quality of life and patient care.

The present study tried to publicize the different variables that may be associated with sleep disorders and consequently the health problems that they can generate in people. It is essential to make proposals to improve the quality of life of nurses to help them to increase their self-esteem and sleep quality, regulate negative emotions, and reduce stress.

Finally, nurses could improve the quality of their sleep by developing organizing strategies so that they can achieve their professional objectives.

## Figures and Tables

**Table 1:** Descriptive statistics of factors of eating, physical exercise, emotional intelligence, and self-esteem, and coefficients of correlation with the sleep quality index (*N* = 1094).

		Mean	*SD*	Correlation PSQI	CI 95%
Three-Factor Eating Questionnaire (R18)	Uncontrolled eating	17.34	5.86	0.17 ***	0.112, 0.227
	Emotional eating	5.74	2.49	0.19 ***	0.132, 0.246
	Restricted eating	16.04	4.56	0.04	−0.019, 0.099
Goal Content for Exercise Questionnaire (GCEQ)	Social affiliation	13.96	5.60	−0.01	−0.069, 0.049
	Image	17.76	4.86	0.06 *	0.001, 0.119
	Management of health	21.72	4.47	−0.00	−0.059, 0.059
	Social recognition	10.91	5.33	0.03	−0.029, 0.089
	Skill development	17.93	5.23	−0.03	−0.089, 0.029
Brief Emotional Intelligence Inventory (EQ–i–M20)	Intrapersonal	9.86	2.87	−0.05	−0.109, 0.009
	Interpersonal	11.65	2.04	−0.01	−0.069, 0.049
	Stress management	12.48	2.23	−0.14 ***	−0.198, −0.081
	Adaptability	11.11	2.13	−0.06 *	−0.119, −0.001
	Mood	11.75	2.33	−0.20 ***	−0.256, −0.142
Rosenberg Self-esteem Questionnaire	Self-esteem	32.45	4.53	−0.23 ***	−0.285, −0.173

Note: PSQI = *Pittsburgh Sleep Quality Index*, ^*^ *p* < 0.05; ^***^ *p* < 0.001.

**Table 2:** Stepwise multiple linear regression model (*N* = 1094).

**Model**	***R***	***R*^2^**		**Corrected *R*^2^**		**Change statistics**							**Durbin Watson**	
						**Std error of estimation**		**Change in *R*^2^**	**Change in *F***		**Sig. of change in *F***			
1 ^(a)^	0.23	0.05		0.05		2.82		0.05	62.29		0.000		1.91	
2 ^(b)^	0.27	0.07		0.07		2.79		0.01	21.90		0.000			
3 ^(c)^	0.29	0.09		0.08		2.77		0.01	18.97		0.000			
4 ^(d)^	0.31	0.09		0.09		2.76		0.00	7.70		0.005			
**Model 4**			**Non-standardized coefficients**				**Standardized coefficients**	***t***	**Sig.**	**CI 95%**			**Collinearity**	
			***B***		**Std. Error**		**Beta**			**Lower limit**		**Upper limit**	**Tol.**	**VIF**
(Constant)			8.89		0.88			10.06	0.000	7.059		10.561		
Self-esteem			−0.11		0.02		−0.17	−5.73	0.000	−0.153		−0.076	0.89	1.11
Emotional eating			0.14		0.03		0.12	4.21	0.000	0.079		0.217	0.91	1.08
Age			0.05		0.01		0.13	4.53	0.000	0.033		0.083	0.99	1.00
Stress management			−0.11		0.04		−0.08	−2.77	0.005	−0.188		−0.033	0.91	1.09

Note: (**a**) Self-esteem; (**b**) Self-esteem, Emotional eating; (**c**) Self-esteem, Emotional eating, Age; (**d**) Self-esteem, Emotional eating, Age, Stress management. VIF = variance inflation factor.

**Table 3:** Stepwise multiple linear regression model (sleep quality) according to marital status.

DIVORCED/SEPARATED	**Model**	***R***				***R*^2^**			**Corrected *R*^2^**				**Change statistics**																				**Durbin Watson**		
													**Std error of estimation**			**Change in *R*^2^**							**Change in *F***				**Sig. of change in F**								
	1	0.65				0.43			0.40				2.64			0.43							16.69				0.000						2.34		
	2	0.73				0.53			0.49				2.44			0.10							4.84				0.039								
	3	0.80				0.64			0.58				2.20			0.10							5.77				0.026								
	**Model 1**						**Non-standardized coefficients**								**Standardized coefficients**				***t***			**Sig.**				**CI 95%**					**Collinearity**				
							***B***				**Std. Error**				**Beta**											**Lower limit**		**Upper limit**			**Tol.**			**VIF**	
	(Constant)						18.00				4.37								4.11			0.001				8.882		27.137							
	Emotional eating						0.49				0.17				0.43				2.88			0.009				0.136		0.847			0.77			1.28	
	Image						−0.42				0.14				−0.39				−2.86			0.010				−0.727		−0.114			0.93			1.07	
	Mood						−0.47				0.19				−0.36				−2.40			0.026				−0.879		−0.062			0.79			1.25	
SINGLE	**Model**	***R***		***R*^2^**					**Corrected *R*^2^**				**Change statistics**																				**Durbin Watson**		
													**Std error of estimation**			**Change in *R*^2^**							**Change in *F***				**Sig. of change in F**								
	1	0.23		0.05					0.05				2.76			0.05							32.90				0.000						1.98		
	2	0.26		0.07					0.06				2.75			0.01							7.59				0.006								
	**Model 2**		**Non-standardized coefficients**									**Standardized coefficients**						***t***		**Sig.**					**CI 95%**							**Collinearity**			
			***B***					**Std. Error**				**Beta**													**Lower limit**					**Upper limit**		**Tol.**			**VIF**
	(Constant)		9.51					1.06										8.96		0.000					6.914					11.396					
	Self-esteem		−0.14					0.02				−0.22						−5.50		0.000					−0.203					−0.098		0.99			1.00
	Age		0.05					0.01				0.11						2.75		0.006					0.019					0.114		0.99			1.00
MARRIED/STABLE RELATIONSHIP	**Model**	***R***				***R*^2^**			**Corrected *R*^2^**				**Change statistics**																				**Durbin Watson**		
													**Std error of estimation**			**Change in *R*^2^**							**Change in *F***				**Sig. of change in F**								
	1	0.25				0.06			0.06				2.80			0.06							35.26				0.000						1.88		
	2	0.31				0.10			0.09				2.75			0.03							19.28				0.000								
	3	0.32				0.10			0.10				2.74			0.00							3.86				0.050								
	**Model 3**				**Non-standardized coefficients**									**Standardized coefficients**			***t***				**Sig.**			**CI 95%**								**Collinearity**			
					***B***					**Std. Error**				**Beta**										**Lower limit**					**Upper limit**			**Tol.**			**VIF**
	(Constant)				9.69					1.07							9.06				0.000			7.593					11.796						
	Mood				−0.25					0.05				−0.20			−4.67				0.000			−0.365					−0.149			0.93			1.06
	Uncontrolled eating				0.08					0.02				0.17			3.83				0.000			0.040					0.123			0.90			1.10
	Stress management				−0.11					0.05				−0.08			−1.96				0.050			−0.228					0.000			0.90			1.10

Note: VIF = variance inflation factor.
